# The effect of rosuvastatin on thromboinflammation in the setting of acute coronary syndrome

**DOI:** 10.1007/s11239-014-1142-x

**Published:** 2014-10-12

**Authors:** Travis R. Sexton, Eric L. Wallace, Tracy E. Macaulay, Richard J. Charnigo, Virgilio Evangelista, Charles L. Campbell, Alison L. Bailey, Susan S. Smyth

**Affiliations:** 1 The Gill Heart Institute, University of Kentucky, BBSRB B201, Lexington, KY 40506 USA; 2 Mario Negri Sud, Santa Maria Imbaro, Chieti Italy

**Keywords:** Acute coronary syndrome, Acute myocardial infarction, Platelets, Statin, Thromboinflammation

## Abstract

In patients with acute coronary syndromes (ACS), early therapy with high-dose statins may reduce short-term adverse clinical outcomes. The mechanisms responsible are not known but could involve anti-inflammatory or anti-thrombotic effects. Compelling evidence from experimental models and clinical studies suggests that the interplay between inflammatory and thrombotic systems, typified by platelet–monocyte and platelet–neutrophil interactions, might be a key regulator of ischemic vascular events. The study sought to determine if early, high-dose administration of the HMG-CoA reductase inhibitor rosuvastatin in the setting of ACS exerts beneficial vascular effects by reducing, and inhibiting biomarkers of thromboinflammation, such as platelet-monocyte and platelet-neutrophil interactions, and biomarkers of myocardial necrosis. A total of 54 patients presenting with ACS within 8 h of symptom onset were randomized to rosuvastatin 40 mg or placebo. Rosuvastatin significantly reduced interactions between platelets and circulating neutrophils (*P* = 0.015) and monocytes (*P* = 0.009) within 24 h. No significant effects were observed on platelet aggregation or plasma levels of PF4, sP-selectin, or sCD40L, whereas significant reductions of RANTES occurred over time in both treatment groups. Plasma levels of myeloperoxidase (MPO) declined more rapidly with rosuvastatin therapy than placebo. In a subset of patients with normal cardiac necrosis biomarkers at randomization, rosuvastatin therapy was associated with less myocardial damage as measured by troponin-I or CK-MB. Early administration of high-dose statin therapy in patients with ACS appears to improve biomarkers of inflammation within 8 h, which may translate into fewer ischemic events.

**Electronic supplementary material:**

The online version of this article (doi:10.1007/s11239-014-1142-x) contains supplementary material, which is available to authorized users.

## Introduction

Platelets play a central role in the pathogenesis of acute coronary syndromes (ACS) and in thrombotic complications of percutaneous coronary intervention (PCI). Anti-platelet therapy is a mainstay of the initial management of patients with non-ST-segment elevation (NSTE)-ACS, ST-segment elevation myocardial infarction (STEMI), and those undergoing PCI [1]. However, despite the general use of anti-platelet agents in ACS, recurrent ischemic events occur in a substantial number of patients. ACS is accompanied by an intense inflammatory response. Compelling evidence from experimental models and clinical studies suggests that interplay between the inflammatory and thrombotic systems might be a key regulator of ischemic vascular events.

Platelet–leukocyte interactions offer an important mechanistic link between the inflammatory and thrombotic systems. Activated platelets bind to circulating leukocytes and recruit them to sites of vascular injury and to thrombus. Experiments in preclinical models indicate that the interactions contribute to local, downstream, and systemic injury responses [2]. In both ACS and PCI, high levels of circulating platelet–leukocyte heterotypic aggregates correlate with markers of myocardial necrosis. Moreover, interventions to block the interactions may reduce ischemic injury. For example, in the recently reported SELECT-ACS trial, the P-selectin antagonist inclacumab, which blocks the ability of platelets to bind to leukocytes [3], reduced cardiac biomarkers of necrosis (troponin-I and creatine kinase-MB [CK-MB]) in patients with ACS [4]. Such findings suggest that interactions between platelets and leukocytes may contribute to ischemic tissue injury in the setting of ACS.

In this study, we sought to determine if administration of a high-dose of the 3-hydroxy-3-methylglutaryl coenzyme A (HMG-CoA) reductase inhibitor (statin) rosuvastatin early in the setting of ACS would exert beneficial effects by reducing thromboinflammation, specifically platelet interactions with monocytes and neutrophils. We selected rosuvastatin because of its ability to lower inflammatory markers in addition to low-density lipoprotein (LDL)-cholesterol. In the JUPITER trial [5], which examined rosuvastatin as primary prevention in patients with increased levels of high-sensitivity C-reactive protein (hs-CRP), the largest benefit occurred in subjects who had lowering of both hs-CRP and LDL-cholesterol levels [6]. Several studies have suggested that the benefits of high dose statin therapy may begin prior to any significant blood cholesterol lowering effect [7, 8]. In these trials, rosuvastatin or atorvastatin reduced peri-procedural cardiac necrosis in stable coronary artery disease and ACS even when administered less than 24 h prior to the procedure [9–12].

To provide mechanistic insight into the actions of early, high dose statin therapy, we enrolled ACS patients who presented within 8 h of symptom onset and randomized them to receive a 40 mg dose of rosuvastatin or placebo and monitored monocyte–platelet and neutrophil–platelet aggregates, markers of platelet activation and inflammation, within the first 24 h. Our findings suggest that administration of high-dose rosuvastatin early in the course of ACS reduces interactions of platelets with monocytes and neutrophils within 8 h and thereby exert a beneficial effect on thromboinflammatory pathways.

## Materials and methods

### Patients and study design

The early use of rosuvastatin in acute coronary syndromes: targeting platelet–leukocyte interactions (AVATAR) trial enrolled patients presenting to the University of Kentucky hospitals with (1) cardiac ischemia within the last 8 h, (2) biomarker evidence of cardiac ischemia and/or (3) electrocardiographic evidence of cardiac ischemia. At screening, patients 18–80 years of age had to either not be taking a statin medication or be on a low dose of a statin (defined as pravastatin ≤40 mg, simvastatin ≤20 mg, or atorvastatin ≤10 mg). Supplemental Table 1 lists additional inclusion and exclusion criteria.

This was an investigator-initiated trial designed by the investigators (www.clinicaltrials.gov, trial NCT01241903). The institutional review board at the University of Kentucky approved the protocol, and all patients provided written informed consent before entering the trial. Patients were enrolled in a double-blind manner and were randomized to high-dose rosuvastatin (40 mg) or a placebo given immediately upon consent. All patients received rosuvastatin 20 mg after the first hospital day and daily thereafter for 30 days. Blood was sampled at baseline, prior to randomization, and at approximately 8 and 24 h following the initial dose. Study follow-up occurred daily while in hospital and at 30 days after enrollment. Major adverse cardiovascular events were collected at 30 days.

### Flow cytometry

See supplemental materials for details.

### Platelet aggregation

See supplemental materials for details.

### Biomarker assays

See supplemental materials for details.

### Clinical outcomes

See supplemental materials for details.

### Statistical analyses

See supplemental materials for details.

## Results

### Patient characteristics

From November 29, 2011, through July 24, 2013, 54 patients were enrolled and 53 received either placebo (*n* = 26) or a 40 mg dose of rosuvastatin (*n* = 27) at study entry. One enrolled patient refused to take the initial dose of study medication and was removed from further analysis. Table 1 lists demographic and baseline characteristics of the patients and Table 2 lists cardiovascular medications that patients received prior to enrollment and in the 24 h following randomization. No significant differences in demographics or cardiovascular medication use were noted between the two groups. Table 3 lists clinical features of the patients. Overall, 37 (70 %) of the subjects had a non-ST-segment elevation myocardial infarction (NSTEMI) or unstable angina (UA), and 17 (30 %) of the patients suffered a STEMI. Of the 26 patients randomized to the placebo group, 10 had STEMI and 16 NSTEMI/UA. Of the 27 patients randomized to the rosuvastatin group, 7 had STEMI and 20 NSTEMI/UA. Forty six of the patients (86.8 %) underwent PCI while in hospital. The average time from hospital admission to PCI was 498 ± 156 min for the placebo group (*n* = 25) and 429 ± 136 for rosuvastatin group (*n* = 21; *P* = 0.744). 42.3 % of the patients in the placebo group and 48 % in the rosuvastatin group underwent PCI prior to the 8 h blood draw. No significant differences were observed in clinically obtained troponin-I levels between the placebo and rosuvastatin groups at near baseline or approximately 24 h after randomization. Additionally, platelet and white blood cell counts at the time of study enrollment and at 24 h were similar between the two groups.

**Table 1:** Baseline characteristics of AVATAR subjects

	Placebo	Rosuvastatin	*P* value
Demographics			
Men	17 (65 %)	16 (59 %)	0.779
Women	9 (35 %)	11 (41 %)	0.779
Age	52.8 (10.7)	57.2 (10.4)	0.142
Caucasian	22 (85 %)	24 (89 %)	0.704
African American	3 (12 %)	3 (11 %)	1.000
Hispanic	1 (3 %)	0 (0 %)	0.491
Cardiovascular History			
Hypertension	18 (69 %)	17 (63 %)	0.773
History of smoking	21 (81 %)	23 (85 %)	0.728
Hyperlipidemia	19 (73 %)	19 (70 %)	1.000
Diabetes	5 (19 %)	7 (26 %)	0.745
Family history of CHD	15 (58 %)	14 (52 %)	0.785
Prior MI	9 (35 %)	5 (19 %)	0.224
Cardiovascular			
Ejection fraction, (%)	49.5 (10.4)	55.4 (7.9)	0.056

Data are *n* (%) or mean (SD)

*P* values for the qualitative variables (sex, race, cardiovascular history) were calculated using Fisher’s Exact test

*P* values for quantitative variables were calculated with a two-sample *t* test

*CHD* coronary heart disease, *MI* myocardial infarction

**Table 2:** Baseline and 24 h medications for AVATAR subjects

	Placebo	Rosuvastatin	*P* value
Medication prior to dosage			
Aspirin	24 (92 %)	24 (89 %)	1.000
P2Y12 inhibitor	22 (85 %)	23 (85 %)	1.000
Beta blocker	13 (50 %)	10 (37 %)	0.412
ACE inhibitor	13 (50 %)	7 (26 %)	0.093
GPI	4 (15 %)	3 (11 %)	0.704
Heparin	19 (73 %)	14 (52 %)	0.158
Bivalirudin	0 (0 %)	1 (4 %)	1.000
Medication within 24 h following dosage			
Aspirin	24 (92 %)	26 (96 %)	0.610
P2Y12 inhibitor	25 (96 %)	25 (93 %)	1.000
Beta blocker	17 (65 %)	22 (81 %)	0.224
ACE inhibitor	16 (62 %)	14 (52 %)	0.583
GPI	4 (15 %)	3 (11 %)	0.704
Heparin	19 (73 %)	20 (74 %)	1.000
Bivalirudin	2 (8 %)	1 (4 %)	0.610

Data are *n* (%) or mean (SD)

*P* values were calculated using Fisher’s Exact test

*GPI* glycoprotein IIb/IIIa inhibitor

**Table 3:** Clinical characteristics of patients

	Placebo	Rosuvastatin	*P* value
ACS			
STEMI	10 (38 %)	7 (22 %)	0.387
NSTEMI/UA	16 (62 %)	20 (78 %)	0.387
Hemogram			
Baseline platelet count	231 ± 48	220 ± 54	0.462
24 h platelet count	215 ± 55	195 ± 47	0.234
Baseline WBC	10.1 ± 0.7	9.9 ± 0.7	0.871
24 h WBC	9.4 ± 0.7	9.4 ± 0.7	0.931
Cardiac Necrosis			
Baseline troponin	0.45 (0.09–2.60)	0.47 (0.08–3.11)	0.907
Peak troponin	9.14 (0.95–35.37)	6.00 (0.12–36.26)	0.478

Data are presented as *n* (%), mean ± SD, or median (25th–75th percentile). STEMI, NSTEMI, and unstable angina (UA) were determined by the attending physician based on ECG and cardiac necrosis biomarker lab results. *P* values were calculated using Fisher’s Exact Test for qualitative variables, a two-sample *t* test for approximately normally distributed quantitative variables, and a Mann–Whitney rank sum test for other quantitative variables

### Effect of early administration of high-dose rosuvastatin on the percentage monocytes and neutrophils with associated platelets

**Fig. 1:**
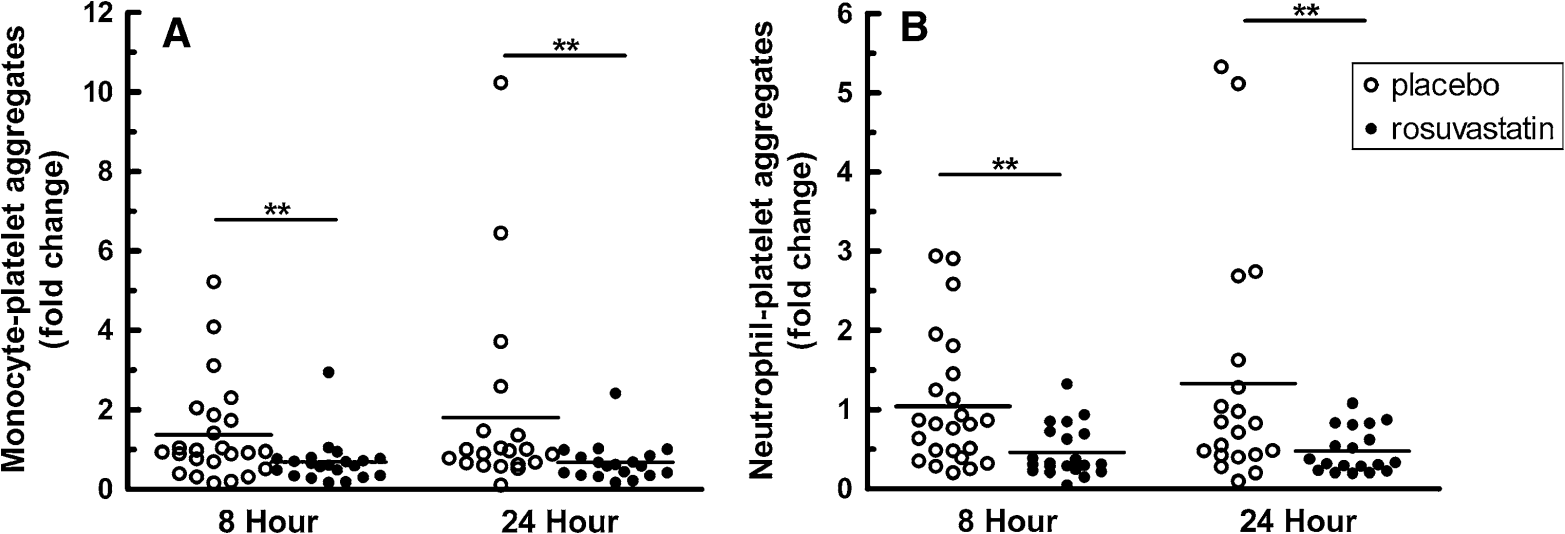
Platelet–monocyte and platelet–neutrophil aggregates in ACS patients randomized to rosuvastatin or placebo. The fold change from baseline of monocytes with attached platelets for each subject is plotted at 8 and 24 h following randomization (**a**). **b** displays the fold change of neutrophils with attached platelets from baseline. Overall significance between groups over 24 h is indicated in Table 4. Significance between the groups at time points are indicated by (**). Statistical significance was ascertained using a linear mixed model and is indicated in Table 4

Early administration of high dose rosuvastatin resulted in a statistically significant reduction in circulating monocyte–platelet aggregates over the first 24 h (*P* = 0.0029). At the time of study enrollment (baseline), the percentage of monocytes with associated platelets was 65.5 ± 6.4 % (mean ± SEM) in the rosuvastatin group and 49.2 ± 3.6 % in the placebo group (*P* = 0.132 after Bonferroni adjustment; Table 4). At 8 h, a striking 25.3 % absolute reduction in average monocyte–platelet aggregates occurred with rosuvastatin, whereas the placebo group had a modest 3.1 % decrease (*P* = 0.0039 for difference between doses from baseline to 8 h, Table 4). No further decline occurred at 24 h, when monocyte–platelet aggregates averaged 40.7 ± 5.6 % with rosuvastatin and 51.6 ± 6.2 % with placebo (*P* = 0.0029 for difference between doses from baseline to 24 h, Table 4). Figure 1a presents the individual data normalized to baseline (fold change where 1.0 equals baseline value) and demonstrates an overall reduction in the proportion of monocytes with platelets in patients who received early, high dose rosuvastatin.

**Table 4:** Monocyte–platelet and neutrophil– platelet interactions are decreased significantly following treatment of a high-dose rosuvastatin

	Baseline % (SE)	*P* value*	8 h % (SE)	*P* value^†^	24 h % (SE)	*P* value^†^	Main finding^‡^
Monocyte–platelet							
Placebo	49.2 (3.6)	0.132	46.1 (6.4)	0.004	51.6 (6.2)	0.003	0.009
Rosuvastatin	65.5 (6.4)		40.2 (4.6)		40.7 (5.6)		
Neutrophil–platelet							
Placebo	23.1 (3.5)	0.197	18.6 (3.9)	0.009	18.1 (3.9)	0.033	0.015
Rosuvastatin	31.7 (4.4)		12.8 (2.1)		13.3 (2.2)		

^*^Linear mixed model comparing treatment groups on baseline values, with Bonferroni adjustment

^†^ Linear mixed model comparing treatment groups on the change from baseline, with Bonferroni adjustment

^‡^ Linear mixed model comparing treatment groups overall, across all time points

Analysis of neutrophil–platelet aggregates demonstrated a significant lowering in the rosuvastatin group over the first 24 h (*P* = 0.0145, Table 4). At baseline, 31.7 ± 4.4 % of the neutrophils had attached platelets in the rosuvastatin group versus 23.1 ± 3.5 % in the placebo group. At 8 h, the average percentage of circulating neutrophils with adherent platelets was markedly lower in the rosuvastatin group at 12.8 ± 2.1 (18.9 % absolute reduction) but only slightly lower in the placebo group at 18.6 ± 3.9 (4.5 % absolute reduction) (*P* = 0.0029 for difference between doses from baseline to 8 h). Average percentages were similar at 24 h, when the neutrophil–platelet aggregates were 13.3 ± 2.2 % in the rosuvastatin group and 18.1 ± 3.9 % in the placebo group (*P* = 0.0109 for difference between doses from baseline to 24 h). Figure 1b displays the fold change in neutrophil–platelet aggregates relative to baseline.

In addition to the primary analysis, an additional analysis was performed by type of event at presentation (NSTEMI/UA and STEMI). In NSTEMI patients, a significant difference in platelet-neutrophil aggregates, but not platelet-monocyte aggregates, occurred at 24 h in the two groups. STEMI patients had significant difference in the decline over 24 h in platelet-monocyte aggregates but not platelet-neutrophil aggregates between the groups (Supplemental Fig. 1 and Supplemental Table 2).

In a subset of patients (*n* = 37), the total number of monocyte–platelet and neutrophil–platelet aggregates per μl of blood was determined (Supplemental Table 3). No significant differences were observed between the rosuvastatin and placebo groups at baseline in the numbers of monocyte–platelet or neutrophil–platelet aggregates per μl blood. At 8 h and 24 h after rosuvastatin therapy, significant declines occurred in the total number of neutrophil–platelet aggregates/μl blood (*P* = 0.0021 and *P* = 0.0052 for 8 and 24 h, respectively). A non-significant trend was observed in monocyte–platelet aggregates/μl blood (*P* = 0.0785) in the rosuvastatin group at 24 h. No significant changes from baseline were observed in the placebo groups for any aggregate type at 8 or 24 h.

### Effect of early high-dose rosuvastatin on platelet function in patients with ACS

**Fig. 2:**
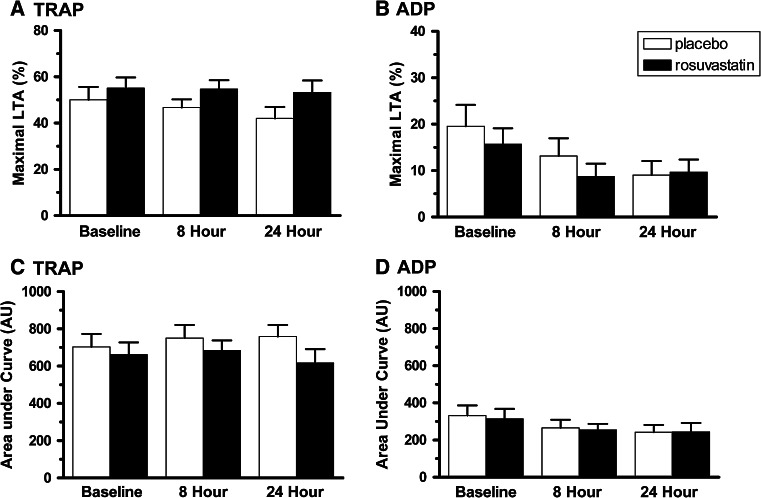
TRAP- or ADP-induced platelet aggregation in ACS patients randomized to rosuvastatin or placebo. Maximum platelet aggregation was measured by light transmission in PRP in response to 15 μM TRAP (**a**) and in response to 5 μM ADP (**b**). Area under the curve values in the Multiplate assay with TRAP (**c**) or ADP (**d**) as an agonist. Values are presented as the mean ± SD for the groups at the indicated times. Statistical significance of change from baseline for each group was determined using a paired *t* test

Several studies have suggested that statins influence platelet activity in vitro, and the effect of rosuvastatin on leukocyte–platelet aggregates could be the result of inhibition of platelet function. TRAP- and ADP- induced platelet aggregation was therefore monitored prior to and up to 24 h following randomization to rosuvastatin or placebo using both light transmission aggregometry (LTA) and multiple electrode aggregometry (MEA). No significant differences in TRAP-induced LTA were observed between groups, nor were there substantial changes over time (Fig. 2a). At 24 h, ADP-induced LTA was lower than baseline in both groups (Fig. 2b), likely due to treatment with P2Y12 antagonists, but there were no significant differences between groups. Similar results were observed with the MEA assays using TRAP (Fig. 2c) and ADP (Fig. 2d) as agonists, although the response to ADP was less apparent in the MEA assay.

The ability to aggregate is one measure of platelet function. We also examined levels of five biomarkers (sCD40L, P-selectin, VEGF, Platelet Factor 4 (PF4), and RANTES), whose concentrations in plasma maybe affected by platelet activation and secretion. The baseline concentrations of sCD40L and RANTES were significantly higher in both the rosuvastatin and placebo groups in comparison with healthy controls, and the values of both declined over 24 h with no significant differences between the two groups (Table 5). There were no significant findings for VEGF, P-selectin, and PF4.

**Table 5:** Biomarkers of platelet activation

Platlet activation	Health control	Placebo			Rosuvastatin		
		Baseline	8 h	24 h	Baseline	8 h	24 h
VEGF (pg/ml)	0.26 (0.00–0.67)	0.99 (0.50–1.59)	1.08 (0.79–1.90)	1.24 (0.59–1.90)	0.71 (0.35–1.59)	0.89 (0.62–1.79)^b^	1.28 (0.44–1.82)
P-Selectin (pg/ml)	11.3 (8.3–19.3)	14.1 (10.2–18.3)	13.1 (9.5–19.7)	14.1 (10.2–18.4)	13.6 (10.9–20.3)	3.6 (11.6–19.1)	12.9 (10.8–18.4)
sCD40L (pg/mL)	207 (165–270)	561 (352–1,299)^a^	528 (223–788)^b^	552 (350–795)^b^	593 (317–1,260)^a^	399 (285–682)	454 (292–837)
RANTES (pg/mL)	147 (138–150)	1,336 (537–5,478)^a^	519 (200–974)^b^	548 (328–1,012)^b^	637 (273–7,862)^a^	343 (184–653)^b^	253 (201–842)
PF4 (pg/mL)	573 (436–619)	418 (164–1,159)	598 (109–2,040)	981 (433–1,501)	853 (132–1,415)	672 (202–1,249)	421 (149–1,740)

Data presented as median (25th–75th percentile)

^a^ When value significantly different from baseline value (Wilcoxon signed-rank test)

^b^ When background value is significantly different from healthy plasma (Mann–Whitney rank sum test)

### Effect of early high-dose rosuvastatin on plasma inflammatory biomarkers in patients with ACS

**Fig. 3:**
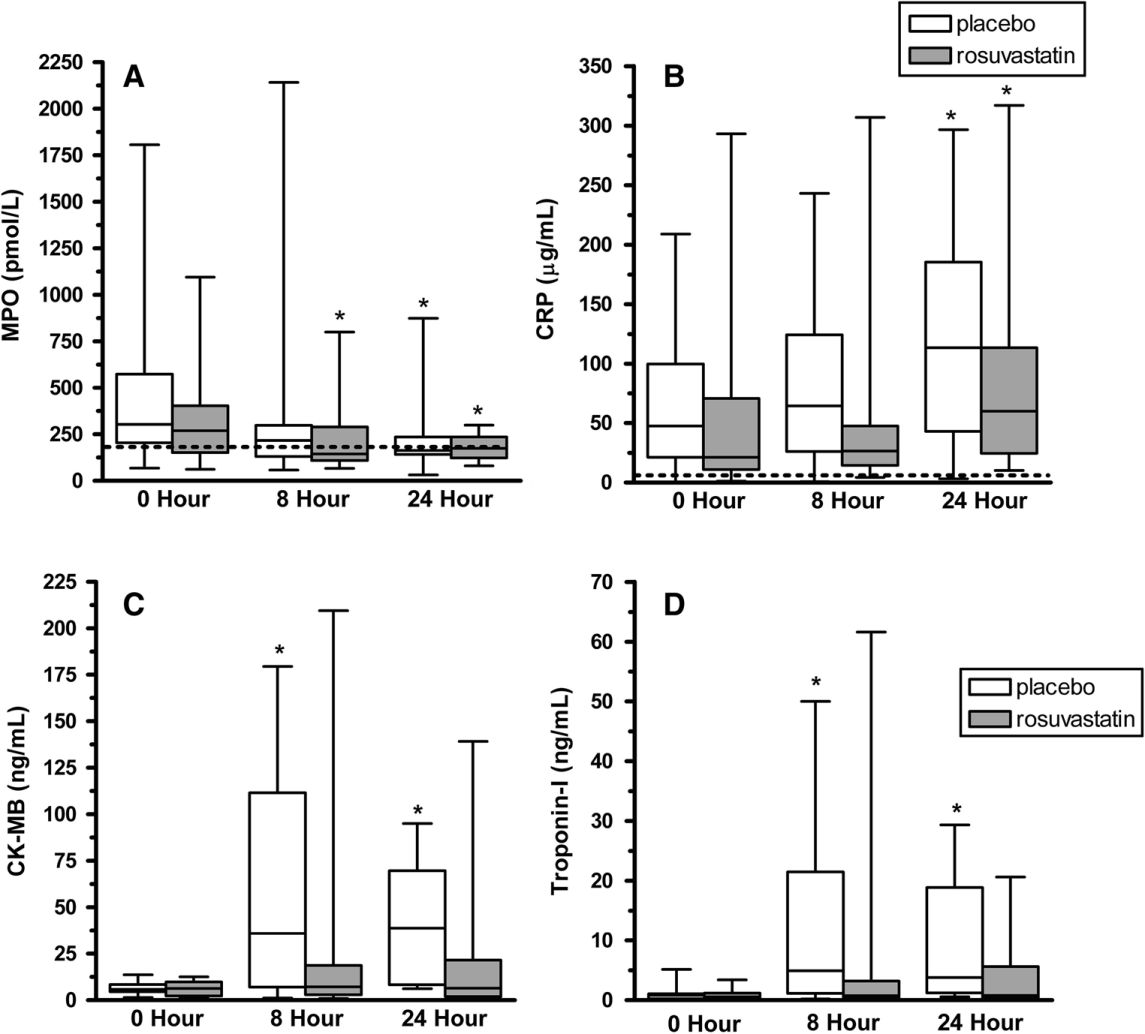
Inflammatory and cardiac necrosis levels in ACS patients randomized to rosuvastatin or placebo. MPO (**a**) and CRP (**b**) levels in subjects randomized to rosuvastatin (*shaded boxes*) and placebo (*white boxes*) at the indicated time points. A subpopulation of patients with low baseline values of CK-MB were identified for subsequent cardiac biomarker analysis. CK-MB levels (**c**) and troponin-I levels (**d**). *Boxes* represent the IQR with the median represented as a solid horizontal line within the box. Whiskers show the extent of the data sets. The *dashed lines* is the MPO and CRP level in pooled plasma from healthy donors. Statistical significance of change from baseline for each group was determined using a Wilcoxon signed rank test. *P* values of less than 0.05 are indicated by (*)

During vascular inflammation, myeloperoxidase (MPO) is released from leukocytes, particularly neutrophils. Interactions with platelets may promote the release of MPO from neutrophils [13], and the decline in neutrophil-associated MPO in patients with acute MI correlates with an increase in circulating leukocyte–platelet aggregates [13, 14]. Importantly, elevated levels of MPO in patients with chest pain predict ischemic cardiovascular events. We therefore examined MPO in study subjects and results are presented in Fig. 3a and Table 6. At baseline, MPO levels were higher in both groups than in healthy controls. The rosuvastatin group (*P* = 0.027), but not the placebo (*P* = 0.067), group had a significant decrease from baseline to 8 h. By 24 h following randomization MPO was also significantly lower in the placebo group (*P* = 0.005) and remained significantly lower from baseline in the rousuvastatin group (*P* = 0.015). A similar trend was seen with CRP (Fig. 3b), which increased from baseline to 8 h in the placebo group (*P* = 0.050) but not in the rosuvastatin group (*P* = 0.269). Both groups had significant increases in plasma CRP 24 h following randomization.

**Table 6:** Biomarkers of inflammation

Inflammation	Healthy control	Placebo			Rosuvastatin		
		Baseline	8 h	24 h	Baseline	8 h	24 h
MPO (pmol/L)	72 (66–82)	303 (204–575)^a^	217 (130–298)	164 (142–235)^b^	270 (152–404)^a^	145 (110–289)^b^	174 (124–236)^b^
IL-6 (pg/mL)	1.13 (1.02–1.23)	3.19 (1.82–5.90)^a^	6.57 (3.18–9.68)^b^	6.25 (3.22–9.87)	3.50 (1.85–14.30)^a^	6.17 (2.89–18.22)^b^	9.99 (3.52–25.87)^b^
ENA-78 (pg/mL)	18.9 (15.5–22.3)	48.8 (34.3–102.0)^a^	34.5 (27.0–81.3)	42.1 (30.3–67.4)	54.4 (20.5–91.6)	29.9 (2.3–67.7)^b^	29.8 (14.2–83.0)
Fractalkine (pg/mL)	28.5 (9.8–37.1)	61.1 (29.7–146.6)^a^	71.4 (18.0–176.9)	82.1 (31.5–103.4)	54.6 (31.4–87.5)^a^	59.6 (31.4–87.5)	53.2 (27.5–134.9)
MCP-1 (pg/mL)	216 (173–250)	196 (126–268)	189 (126–278)	153 (124–309)	167 (113–224)	166 (116–260)	216 (173–250)
MIP-1α (pg/mL)	11.8 (10.9–12.5)	3.1 (11.1–14.7)	12.6 (10.2–13.4)	11.8 (10.9–14.5)	12.2 (10.5–14.3)	11.1 (9.7–13.5)	11.0 (8.8–12.9)^b^
MIP-1β (pg/mL)	11.7 (10.9–12.5)	18.6 (15.1–23.7)^a^	17.5 (12.8–22.1)	18.3 (14.8–22.1)	19.3 (14.1–29.1)^a^	17.4 (15.1–24.3)	11.8 (10.9–12.5)
NAP2 (pg/mL)	262 (222–312)	1,360 (719–2,358)^a^	538 (358–1,485)^b^	586 (469–972)^b^	1,037 (547–1,990)^a^	418 (241–762)^b^	494 (289–795)^b^
TNF-α (pg/mL)	0.58 (0.53–1.07)	1.09 (0.86–1.83)^a^	0.97 (0.79–1.51)	1.00 (0.83–1.57)	1.16 (0.93–1.51)	1.00 (0.79–1.51)	0.58 (0.53–1.07)

Data presented as median (25th–75th percentile)

^a^ When background value is significantly different from healthy plasma (Mann–Whitney rank sum test)

^b^ When value significantly different from baseline value (Wilcoxon signed-rank test)

Table 6 presents information on additional inflammatory cytokines. At baseline, the levels of IL-6, ENA-78, MIP-1β, and NAP2 were significantly higher in one or both groups than in healthy controls. IL-6 levels significantly increased at 8 h in both groups and continued to rise at 24 h in the rosuvastatin group. ENA-78 and NAP2 decreased at 8 and 24 h in both groups. MIP-1β remained relatively unchanged in the placebo group at 8 h, with a trend towards reduction at 24 h in the rosuvastatin group.

### Cardiac necrosis markers in patients with ACS

While no significant differences were observed in baseline and peak troponin-I levels between the two groups (Table 3), a post hoc sub-analysis was performed in patients who presented with values <3× the upper limit of normal to investigate the effect of rosuvastatin on biomarkers of cardiac necrosis. A total of 24 subjects met this criterion (*n* = 12 in each group). At 8 h, CK-MB levels were elevated significantly from baseline in the placebo group (*P* = 0.007, Fig. 3c) but not in the rosuvastatin group (*P* = 0.176). Similarly, there was significant increase in troponin-I levels in the placebo group (*P* = 0.003, Fig. 3d) but not the rosuvastatin group (*P* = 0.110). At 24 h troponin-I levels remained significantly higher than baseline in the placebo group (*P* = 0.037) but had not increased significantly from baseline in the placebo group (*P* = 0.084). There were no major adverse ischemic events during the hospital stay for any of the enrolled patients. No major bleeding occurred in any subjects. Within the first 30 days, one subject died due to recurrent MI, and one suffered a stroke. Both individuals had been randomized to the placebo group.

## Discussion

Previous studies have indicated that statins may exert protective effects if taken within 24 h prior to PCI in patients with ACS [15], although the mechanism is not understood. In the AVATAR study, we demonstrated that intensive statin therapy may be associated with beneficial effects within 8 h of administration in patients presenting with ACS. Early treatment with rosuvastatin 40 mg lowered the percentage of monocytes and neutrophils with attached platelets within 8 h and was accompanied by a decline in MPO levels. The changes in monocyte–platelet and neutrophil–platelet interactions occurred without a detectable effect of rosuvastatin on platelet aggregation or soluble levels of P-selectin, CD40L, or PF4. Based on subgroup analysis, acute administration of rosuvastatin was associated with reduced biomarkers of cardiac damage within the first 24 h, as has been reported in other settings [16]. Previous studies that observed cardiac necrosis markers following PCI in patients that were pre-loaded with statin demonstrate an acute benefit of statins. Here, we demonstrate acute effects of statins on monocyte–platelet and neutrophil–platelet aggregates in patients presenting with ACS. These findings are also consistent with reports that rosuvastatin reduces platelet–leukocyte aggregate formation in a model of congestive heart failure [17] and blocks postprandial activation of neutrophils [18], although both of these studies examined long term effects of statins, over weeks and did not report immediate effects. The novelty of our study is the identification of an acute effect (<24 h) of statin therapy on biomarkers of thromboinflammation in the setting of ACS.

Statins work by inhibiting the function of HMG-CoA reductase, which, in turn, lowers the de novo synthesis of cholesterol. Rosuvastatin and other members of the class may have pleiotropic effects that are independent of lowering LDL-cholesterol levels. Our findings are consistent with a rapid mechanism of action independent of LDL-cholesterol and suggest that the ability of rosuvastatin, and potentially other statins, to impair platelet–leukocyte interactions could translate into long-term clinical benefit on top of that gained by lowering cholesterol. The AVATAR results are consistent with observations in experimental and in vitro models in which HMG-CoA reductase inhibition attenuated leukocyte–platelet interactions [12, 19]. In vitro, statins reduce the expression of mediators of heterotypic blood cell interactions, such as sCD40, ICAM, and E-selectin [20–22]. Additional mechanisms of effect may include improved endothelial function, decreased oxidative stress, and inhibition thrombogenic responses not measured by platelet aggregation to ADP or thrombin [23].

The precise mechanism by which rosuvastatin reduce platelet-leukocyte interaction remain to be defined. However, much of the data seems to suggest that leukocytes are the cellular targets of the statin. This is in agreement with in vitro studies demonstrating that HMG-CoA reductase inhibitors block Mac-1 activation in monocytes [24]. This is particularly relevant since Mac-1 mediates platelet leukocyte adhesion.

The AVATAR results may also reflect differences in temporal patterns between the rosuvastatin and placebo groups. Although no statistically significant difference was observed in overall monocyte–platelet and neutrophil–platelet aggregates at baseline between the two groups, numerically the average percentage of monocytes and neutrophils with attached platelets was higher at baseline in the individuals randomized to rosuvastatin. If these patients were captured at a different time in the presentation of ACS, more rapid reduction in aggregates could have resulted. In that case, we would anticipate that heterotypic aggregates would continue to decline in the placebo group at 24 h, but they did not. Most of the inflammatory biomarkers declined or remain unchanged within the first 24 h after presentation. The exceptions were IL-6, which was higher at 24 h in the rosuvastatin group at a time when the frequency of monocyte–platelet and neutrophil–platelet aggregates was reduced, and CRP which increased from baseline at 8 h in the placebo group but not in the rosuvastatin group.

In summary, the results of the AVATAR trial indicate that targeting pathways that link inflammation and thrombosis may be a beneficial strategy in patients with ACS. Although the sample size was too small to identify an effect on clinical outcomes, when considered with previously published work that demonstrated a reduction in ischemic and clinical events with early high dose statins that associated with reduced biomarkers of cardiac necrosis, our findings suggest that reducing monocyte–platelet and neutrophil–platelet interactions may contribute to the acute benefit that has been observed. If this is true, high-dose statin therapy should be administered rapidly, similarly to aspirin therapy, in patients presenting with ACS to maximize effects independent of LDL-cholesterol lowering.

## Electronic supplementary material

Below is the link to the electronic supplementary material.

Supplementary material 1 (DOC 774 kb)

Supplementary material 2 (DOC 34 kb)
