# Supplementary material for: The effect of rosuvastatin on thromboinflammation in the setting of acute coronary syndrome
Source: J Thromb Thrombolysis. 2014 Oct 12;39(2):186–95. doi: 10.1007/s11239-014-1142-x (PMC4320305; doi:10.1007/s11239-014-1142-x)
Supplement: Supplementary file 1 — Supplementary material 1 (DOC 774 kb) [file 11239_2014_1142_MOESM1_ESM.doc]

**Supplemental Table 1.**

| **Inclusion Criteria** |
| --- |
| - Men or non-pregnant women must be between 18 and 80 years old. |
| - Subjects must be willing and able to give informed consent. |
| - Subjects must have symptoms of acute coronary syndrome as defined by 2 of the 3:   1)  A history of cardiac-ischemia related symptoms of at least 10 minutes  duration ≤ 8 hours prior to randomized treatment assignment.  2) Concurrent biomarker evidence of cardiac ischemia, as defined by  troponin I or T greater than upper limit of normal (ULN) or creatine  kinase-myocardial band (CK-MB) greater than ULN.  3) Concurrent electrocardiographic evidence of cardiac ischemia, as defined  by new or presumably new ST-segment depression(≥ 1mm) or transient  (<30 min) ST-segment elevation (≥ 1mm) in at least two contiguous  leads. |
| - Statin naïve or currently on low dose statin (simvastatin 20 mg, pravastatin 40 mg, or atorvastatin 10 mg). |
| **Exclusion Criteria** |
| - Age < 18 years or > 80 years - Pregnancy |
| - Use of rosuvastatin in the last month |
| - GFR (estimated) < 30 ml/min |
| - Hemodialysis |
| - History of liver failure |
| - Unexplained liver function abnormalities |
| - Current or planned use of cyclosporine or gemfibrozil |
| - Sepsis |
| - Hypotension |
| - Dehydration |
| - Trauma |
| - Severe metabolic, endocrine or electrolyte abnormality |
| - Recent (within the last 2 weeks) or planned (in the next month) major surgery |
| - HIV/AIDS with current or planned use of HIV protease inhibitors |

**Supplemental Table 2. Monocyte-Platelet and Neutrophil-Platelet** Aggregates in the STEMI and NSTEMI subgroups.

| **NSTEMI/UA** | | **Baseline**  % (SEM) | **p**  **value^*^** | **8 Hour**  % (SEM) | **p**  **value^†^** | **24 Hour**  % (SEM) | **p**  **value^†^** | **Main Finding‡** |
| --- | --- | --- | --- | --- | --- | --- | --- | --- |
| **Monocyte-Platelet** |  | | NA |  | NA |  | NA | 0.1796 |
| Placebo | 50.3 (7.2) | |  | 44.3 (7.8) |  | 52.3 (7.4) |  |  |
| Rosuvastatin | 65.9 (7.8) | |  | 45.7 (5.8) |  | 35.6 (7.2) |  |  |
| **Neutrophil-Platelet** |  | | 0.4173 |  | 0.0273 |  | 0.0372 | 0.0296 |
| Placebo | 21.0 (3.8) | |  | 20.0 (5.5) |  | 21.1 (6.2) |  |  |
| Rosuvastatin | 35.7 (6.5) | |  | 14.2 (3.3) |  | 13.1 (2.6) |  |  |
| **STEMI** | **Baseline**  % (SEM) | | **p value^*^** | **8 Hour**  % (SEM) | **p value^†^** | **24 Hour**  % (SEM) | **p value^†^** | **Main Finding‡** |
| **Monocyte-Platelet** |  | | 0.6300 |  | 0.0024 |  | 0.051 | 0.0057 |
| Placebo | 47.5 (13.1) | |  | 49.0 (11.7) |  | 50.5 (11.4) |  |  |
| Rosuvastatin | 86.4 (3.3) | |  | 19.9 (7.9) |  | 55.2 (9.8) |  |  |
| **Neutrophil-Platelet** |  | | NA |  | NA |  | NA | 0.2877 |
| Placebo | 26.7 (7.0) | |  | 16.2 (5.1) |  | 13.5 (2.7) |  |  |
| Rosuvastatin | 34.9 (7.2) | |  | 10.7 (1.3) |  | 11.9 (3.5) |  |  |
| * linear mixed model comparing treatment groups on baseline values, with Bonferroni adjustment | | | | | | | | |
| † linear mixed model comparing treatment groups on the change from baseline, with Bonferroni adjustment | | | | | | | | |
| ‡ linear mixed model comparing treatment groups overall, across all time points. | | | | | | | | |
| NA - not applicable. No Post-hoc tests since main finding is not significant | | | | | | | | |

**Supplemental Table 3. The absolute number of aggregates is reduced acutely in ACS patients randomized to rosuvastatin.**

|  | **Placebo** | **p value** | **Rosuvastatin** | **p value** |
| --- | --- | --- | --- | --- |
| **Platelet-Monocyte Aggregates** |  |  |  |  |
| **Baseline** | 134 (52-216) | 0.172^ | 220 (106-360) | 0.172^ |
| **8 hours** | 139 (68-419) | 0.475* | 181 (73-296) | 0.127* |
| **24 hours** | 217 (114-293) | 0.333* | 117 (83-244) | 0.079* |
| **Platelet-Neutrophil Aggregates** |  |  |  |  |
| **Baseline** | 815 (313-2061) | 0.387^ | 1476 (372-1995) | 0.387^ |
| **8 hours** | 624 (372-1233) | 0.382* | 414 (232-671) | 0.002* |
| **24 hours** | 774 (357-1114) | 0.333* | 421 (236-801) | 0.005* |
| Data presented in median number of aggregates / μL whole blood (25^th^ – 75^th^ percentile) | | |  |  |
| *Wilcoxon signed rank test was used to compare values to baseline in each group | | | |  |
| ^Mann Whitney test was used to compare baselines between groups | | | |  |

**
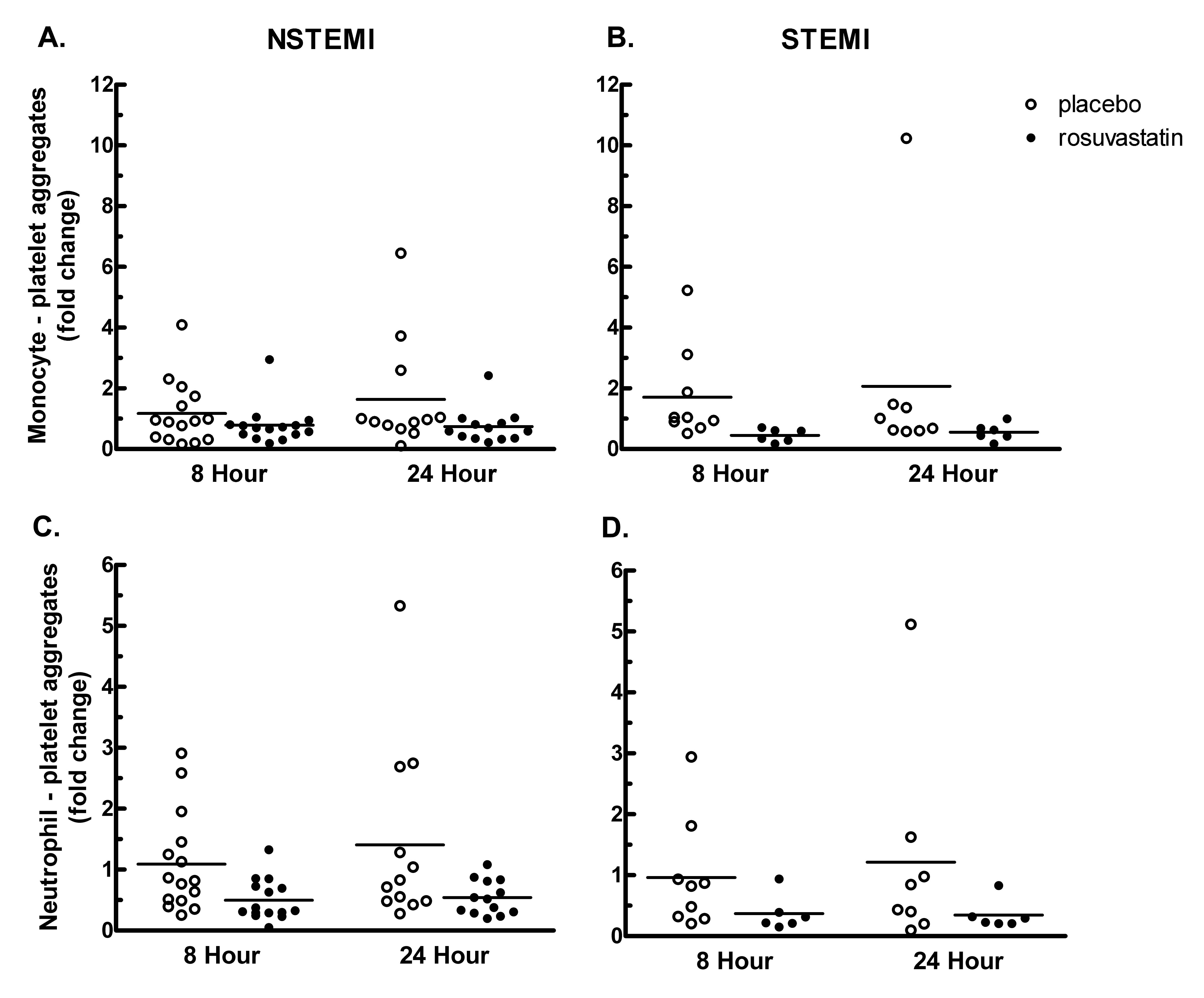
**

**Supplemental Figure 1. Monocyte – platelet and neutrophil – platelet aggregates in NSTEMI and STEMI patients.** Fold-change in monocyte - platelet aggregates in NSTEMI (A) and STEMI (B) patients; neutrophil – platelet aggregates in NSTEMI (C) and STEMI (D) patients. Statistical significance was ascertained using a linear mixed model and is indicated in Supplemental Table 3.
