# Supplementary material for: The effect of rosuvastatin on thromboinflammation in the setting of acute coronary syndrome
Source: J Thromb Thrombolysis. 2014 Oct 12;39(2):186–95. doi: 10.1007/s11239-014-1142-x (PMC4320305; doi:10.1007/s11239-014-1142-x)
Supplement: Supplementary file 2 — Supplementary material 2 (DOC 34 kb) [file 11239_2014_1142_MOESM2_ESM.doc]

**Supplemental Methods and Materials**

**Flow Cytometry**

Platelet – leukocyte aggregate analysis was performed by flow cytometry as previously described [^9^](#_ENREF_10). Fresh whole blood was collected in hirudin (Hirudin Blood tubes, diaPharma, West Chester, Ohio) and incubated with leukocyte specific APC-conjugated anti-CD11b/Mac-1 (BD Biosciences, San Jose, California) and platelet specific FITC-conjugated anti-CD42b (BD Biosciences) for 15 minutes at room temperature in the dark. Flow cytometry was performed following red blood cell lysis and fixation (FACS Lysing Solution;BD Biosciences). Typical samples included 50,000 leukocytes. Monocytes and neutrophils were identified by CD11b-positive and the forward-scatter and side-scatter profiles. Where indicated, to determine the number of aggregates per μl of whole blood, a known concentration of CountBright™ absolute counting beads (Invitrogen, Carlsbad, California) was added to prepared sample. FlowJo software version 7.6.5 (TreeStar Inc, Ashland, Oregon) was used for analysis.

**Platelet Aggregation**

Aggregometry of platelet rich plasma (PRP) was performed as previously described using a light transmission aggregometer (Chrono-log Corp, Havertown, Pennsylvania) in PRP prepared from citrated blood [^10^](#_ENREF_11). PRP was incubated at 37°C for 180 seconds prior to addition of either PAR-1 activating peptide (TRAP, 15 µM final concentration) or ADP (5 μM final concentration). The maximal aggregation and slope within the first 3 minutes were calculated using the AGGROLINK software (Chrono-log Corp, Havertown, Pennsylvania). Multiplate impedance analysis was also performed on hirudin-anticoagulated whole blood with the same concentrations of agonist. Blood was incubated in Multiplate cuvettes at 37°C for 120 seconds prior to additions of PAR-1 activating peptide and ADP. Area under the curve (AUC) within 6 minutes of adding agonist was recorded with the Multiplate system (Roche, Basel, Switzerland).

**Biomarker Assays**

Plasma was obtained from CTAD-anticoagulated blood supplemented with a final concentration of 10 μM EDTA prior to centrifugation (3000g x 10 min). Plasma was aliquoted, flash frozen, and stored at -80°C until enrollment was complete. Analytes were measured with MAGPIX multiplex reader, with the exceptions of myeloperoxidase (MPO) and Platelet Factor 4 (PF4), which were quantified with the CardioMPO assay (Cleveland Heart Lab, Cleveland, Ohio) and Duoset CXCL4/PF4 assay (R&D Systems, Minneapolis, MN), respectively. Blood from healthy donors (n = 5) was used to prepare pooled normal plasma and serum, which was run as a control for consistency between the assays and plates.

**Clinical Outcomes**

Rates of major adverse cardiovascular events (cardiovascular death, MI, stroke, recurrent ischemia with re-hospitalization, and urgent coronary revascularization) and bleeding within the first 30 days were determined by patient follow-up and review of electronic medical records.

**Statistical Analyses**

Patients in the two treatment groups (rosuvastatin, placebo) were compared on baseline characteristics, medical profiles, and clinical characteristics using Fisher’s exact test (qualitative variables), a two-sample t test (approximately normally distributed quantitative variables), or a Mann-Whitney rank sum test (other quantitative variables). Neutrophil-platelet and monocyte-platelet interactions were analyzed using a linear mixed model that included treatment (rosuvastatin, placebo) and time (baseline, 8 hr, 24 hr) as categorical explanatory variables and that included random effects to account for correlations among repeated observations on the same individual. If a significant overall difference between treatment groups was obtained, Bonferroni-adjusted post-hoc tests were performed to compare treatment groups at baseline and to compare treatment groups on change from baseline at both 8 hr and 24 hr. Similar linear mixed model analyses were performed separately for STEMI and NSTEMI subgroups as well as to examine ADP- and TRAP-induced aggregation. Biomarkers, which were in many cases not approximately normally distributed, and myeloperoxidase (MPO) were analyzed nonparametrically. More specifically, Wilcoxon signed rank tests were used to compare 8 hr and 24 hr scores to baseline within each treatment group, and Mann-Whitney rank sum tests were used to compare treatment groups to each other and to healthy volunteers at baseline. Selected biomarkers were also examined in subgroups defined by initial levels less than three times the upper limit of normal. Statistical significance was defined by a p-value less than 0.05. Linear mixed models were fit using version 9.3 of SAS software.
